# Lysophosphatidic acid is associated with neuropathic pain intensity in humans: An exploratory study

**DOI:** 10.1371/journal.pone.0207310

**Published:** 2018-11-08

**Authors:** Ken Kuwajima, Masahiko Sumitani, Makoto Kurano, Kuniyuki Kano, Masako Nishikawa, Baasanjav Uranbileg, Rikuhei Tsuchida, Toru Ogata, Junken Aoki, Yutaka Yatomi, Yoshitsugu Yamada

**Affiliations:** 1 Department of Anesthesiology and Pain Relief Center, The University of Tokyo Hospital, Bunkyo-ku, Tokyo, Japan; 2 Department of Pain and Palliative Medicine, The University of Tokyo Hospital, Bunkyo-ku, Tokyo, Japan; 3 Department of Clinical Laboratory Medicine, The University of Tokyo Hospital, Bunkyo-ku, Tokyo, Japan; 4 Department of Molecular and Cellular Biochemistry, Graduate School of Pharmaceutical Sciences, Tohoku University, Sendai, Miyagi, Japan; 5 Department of Rehabilitation for Movement Functions, Research Institute, National Rehabilitation Center for Persons with Disabilities, Tokorozawa, Saitama, Japan; Tokyo Metropolitan Institute of Medical Science, JAPAN

## Abstract

The underlying mechanisms of neuropathic pain remain to be elucidated. Basic animal research has suggested that lysophosphatidic acids, which are bioactive lipids produced by autotaxin from lysophosphatidylcholine, may play key roles in the initiation and maintenance of neuropathic pain. Here, we investigated the clinical relevance of lysophosphatidic acids signaling on neuropathic pain in humans. Eighteen patients who had been diagnosed with neuropathic pain with varied etiologies participated in the study. Cerebrospinal fluid samples were obtained by lumbar puncture and the concentrations of 12 species of lysophosphatidic acids and lysophosphatidylcholine, autotaxin, and the phosphorylated neurofilament heavy subunit were measured. Pain symptoms were assessed using an 11-point numeric rating scale and the Neuropathic Pain Symptom Inventory regarding intensity and descriptive dimensions of neuropathic pain. The total lysophosphatidic acids were significantly associated with both pain intensity and symptoms. 18:1 and 20:4 lysophosphatidic acids in particular demonstrated the most correlations with dimensions of pain symptoms. Autotaxin and the phosphorylated neurofilament heavy subunit showed no association with pain symptoms. In conclusions, lysophosphatidic acids were significantly associated with pain symptoms in neuropathic pain patients. These results suggest that lysophosphatidic acids signaling might be a potential therapeutic target for neuropathic pain.

## Introduction

The International Association for the Study of Pain (IASP) defines neuropathic pain as “pain caused by a lesion or disease of the somatosensory nervous system”. Neuropathic pain is a debilitating condition that commonly impairs activities of daily living and health-related quality of life, and its prevalence is around 7–8% in the general population [1]. Chronic pain can lead to psychological distress (i.e., anxiety and depression), sleep disorders, and interference with work and social activities. Neuropathic pain is also associated with a heavy individual and societal economic burden.

Multiple pathological mechanisms underlying neuropathic pain have been investigated from a variety of standpoints. In molecular biology, various molecules such as lysophospholipids [2], chemokines [3], and purinergic receptors [4, 5] have been proposed to underlie the development and maintenance of neuropathic pain. In systems biology, various nervous systems such as neuron-glial cell interactions [6] and neuroplasticity of descending pain inhibitory pathways [7] have been related to neuropathic pain. As one of these hypotheses, lysophospholipids, particularly lysophosphatidic acid (LPA), are considered as a potential mechanism underlying the development and maintenance of neuropathic pain [8]. LPA is one of several lipid mediators released after tissue injury, and is produced from lysophosphatidylcholine (LPC) by autotaxin. The administration of LPA into the lumbar intrathecal space can not only lead to nerve damage (i.e., demyelination of the spinal dorsal root), but can also cause subsequent neuropathic pain, such as hyperalgesia (exaggerated pain sensations in response to mildly noxious stimuli) and allodynia (pain perception upon innocuous tactile stimuli). Intense pain signals following either LPA administration or spinal nerve ligation lead to LPA production in the spinal cord, which consequently amplifies LPA production through microglia activation. LPA then returns to the dorsal root and causes demyelination and subsequent sprouting, which deteriorates abnormal pain through physical cross-talk between nociceptive and innocuous (tactile) sensory fibers. LPA upregulates voltage-gated Ca alpha-2-delta subunit channels, which enhances pain transmission through A-fibers, and ephrin B1, which enhances excitatory glutamate N-Methyl-D-aspartic acid receptor transmission. This LPA-induced pain-deteriorating mechanism is observed in the spinal dorsal horn, as well as in secondary pain transmission and descending pain inhibitory systems. Thus, LPA induces a ‘feedforward’ mechanism in peripheral to central chronic neuropathic pain [2]. Moreover, LPC and autotaxin mediate both demyelination and neuropathic pain through LPA and LPA receptors [9, 10].

There are several LPA species, which correspond to LPC species, and these differentially activate several kinds of LPA receptors [11]. Among LPA and LPC species, 16:0, 18:0, and 18:1 LPC increase in the spinal dorsal horn after nerve injury [12]; however, only 18:1 LPA converted from 18:1 LPC by autotaxin is involved in the ‘feedforward’ amplification of LPA production and the development of neuropathic pain via LPA1 and LPA3 receptors [13].

We investigated whether cerebrospinal fluid (CSF) concentrations of LPA, LPC, and autotaxin reflect neuropathic pain conditions in humans. We also investigated which molecular species of LPA and LPC have a profound impact on neuropathic pain in human subjects.

## Materials and methods

The study protocol was approved by the Institutional Review Board of the Ethics Committee of The University of Tokyo (approval number: 10516). We invited patients to participate in this study after they were referred to us by our outpatient clinic. All participants provided written informed consent. The three inclusion criteria were as follows: presence of neuropathic pain as diagnosed by the IASP [14]; aged at least 20 years old and able to provide informed consent; and no other neurological or psychological impairments. The details of the IASP neuropathic pain have been described elsewhere [14]. Briefly, patients were diagnosed with neuropathic pain when their pain had a neuroanatomically plausible distribution; there was a history suggestive of a relevant lesion or disease affecting the peripheral and/or central somatosensory nervous system; a distinct neuroanatomically plausible distribution was demonstrated by at least one neurological examination that confirmed negative or positive signs of somatosensory nervous system impairment; and presence of the relevant lesion or disease was demonstrated by at least one confirmatory test (e.g., imaging or electrophysiological studies). We considered the intensity of neuropathic pain as a study-inclusion criterion (i.e., numeric rating scale (NRS) value > 1 on an 11-point NRS). The duration of neuropathic pain was not included as an inclusion criterion, because the time course of LPA and LPC was not fully elucidated. Furthermore, we did not include the location of neuropathic pain, because it was unclear whether the location of a lesion affects LPA, LPC, or autotaxin concentrations in CSF. The three exclusion criteria were as follows: inability to understand the questions in Japanese without any help; inability to provide informed consent; and altered level of consciousness.

All participants provided CSF samples, which were collected by lumbar punctures of the L3/L4 or L4/L5 interspace. Participants were asked to quantify the average and maximum intensity of their neuropathic pain over the last week on an 11-point NRS, where “0” and “10” indicated “no pain” and “pain as bad as you can imagine”, respectively. Neuropathic pain symptoms were assessed with the Japanese version of the NPSI [15, 16], which assesses ten neuropathic symptoms corresponding to five different dimensions (i.e., symptom combinations) on an 11-point NRS. The NPSI dimensions are “burning (superficial) spontaneous pain”, “pressing (deep) spontaneous pain”, “paroxysmal pain”, “evoked pain”, and “paresthesia/dysesthesia”; severity of neuropathic pain can be evaluated based on the nature of pain. Mental status was assessed using the Hospital Anxiety and Depression Scale (HADS), which consists of seven questions regarding anxiety (HADS-A) and depression (HADS-D).

### Laboratory analyses

The CSF samples were mixed and sonicated with a 10-fold volume of methanol and an internal standard. After centrifugation at 21,500 *g, the resulting supernatant was recovered and used for the LC-MS analysis. Then, 20 μl of methanol extract was separated using Nanospace LC (Shiseido) equipped with a C18 CALL PAK ACR column (1.5*250 mm; Shiseido) using a gradient of solvent A (5 mM ammonium formate in water) and solvent B (5 mM ammonium formate in 95% [v/v] acetonitrile). Elution was sequentially ionized using an ESI probe, and the parent ion (m/z 380.2) and the fragment ion (m/z 264.2) were monitored in the positive mode using the Quantum Ultra Triple Quadrupole Mass Spectrometer (Thermo Fisher Scientific). For LPA and LPC molecular species, 12 acyl chains (14:0, 16:0, 16:1, 18:0, 18:1, 18:2, 18:3, 20:3, 20:4, 20:5, 22:5, and 22:6) were monitored respectively. In the present study, we extracted lipids under a neutral condition, and we did not separate 1-acyl-2-lyso- and 2-acyl-1-lyso-phospholipids. A more detailed description of measuring lysophospholipids is available in our previous article [17]. Briefly, we calculated the concentrations of lysophospholipids from the area ratio to the internal standard as follows: 1 μM 17:0 LPA for LPA species or 10 μM 17:0 LPC for LPC species. The autotaxin antigen levels in the CSF were determined using a two-site immunoenzymetric assay with an autotaxin assay reagent and the TOSOH AIA system (TOSOH, Tokyo, Japan) [18].

In addition to LPA, LPC, and autotaxin, we measured the phosphorylated neurofilament heavy subunit (pNF-H), a known biomarker of the severity of neuronal damage. pNF-H is a major structural protein in CNS axons, and increased pNF-H levels in CSF are observed in patients with the lumbar canal stenosis, which reflects severity of clinical symptoms (e.g., intermittent claudication and leg pain) [19]. CSF pNF-H levels were determined using a commercially available enzyme-linked immunosorbent assay kit (Human Phosphorylated Neurofilament H ELISA; BioVendor, Modrice, Czech Republic) according to the manufacturer’s protocol. The CSF samples were diluted threefold before analysis. pNF-H positivity was defined by levels of >70.5 pg/ml [20].

### Statistical analysis

All the data were statistically analyzed using R version 3.4.0 (The R Foundation for Statistical Computing, Vienna, Austria). The results are expressed as mean (standard deviation) or median (interquartile range) as appropriate. Correlations between measurements and clinical symptoms were tested using Spearman’s correlation test, because the normality of many variables, especially the laboratory measurements in the CSF, was rejected by the Kolmogorov-Smirnov test. Because this was an exploratory study to elucidate the role of LPA and others on neuropathic pain in human subjects, we did not conduct any multivariate analyses. P-values less than 0.05 were regarded as statistically significant in all the analyses.

## Results

Eighteen patients (7 men and 11 women, mean age = 67.2 years) were enrolled in this study. Patients’ characteristics are presented in Table 1. Patients had different etiologies of neuropathic pain, including postherpetic neuralgia (n = 2), cervical myelomalacia (n = 2), thoracic myelitis (n = 2), lumbar adhesive arachnoiditis with or without lumbar surgery (n = 5), lumbar radiculopathy (n = 4), chemotherapy-induced peripheral neuropathy (n = 2), and nutritional polyneuropathy (n = 1). Participants presented a wide range of pain severity in this study; Table 1 also shows the maximum and average pain intensity of NRS and the total scores of NPSI for each subject. Although patient J presented relatively low pain intensity (NRS scores), the patient was included in this study to investigate the correlation of symptoms with wide ranges of pain intensity. We also compared the parameters such as pain scores and the CSF measurements between female and male. As shown in Table 2, male patients tended to show higher levels of pain scores (i.e. total NPSI) and LPAs concentrations (i.e. total LPA) than female.

**Table 1:** Characteristics of patients with neuropathic pain.

Subject	Diagnosis	Sex	Age	Body mass index	Disease duration (years)	NRS maximum	NRS average	NPSI total score	Medication (daily dose)
Patient A	Cervical myelomalacia	F	72	22.1	4	5	4	41	Pregabalin (100 mg), duloxetine (25 mg)
Patient B	Post-herpetic myelitis	F	67	22.4	10	9	7	47	Pregabalin (150 mg), carbamazepine (100 mg), imipramine (20 mg), baclofen (30 mg), acetaminophen (1800 mg), muscular relaxants (as needed)
Patient C	Cervical myelomalacia	M	74	21.9	1	8	7	45	Pregabalin (150 mg), tramadol (150 mg)
Patient D	Chemotherapy-induced peripheral neuropathy	M	67	27.9	8	4	4	20	Clomipramine (75 mg)
Patient E	Post-herpetic neuralgia	M	70	19.7	4	9	6	89	Pregabalin (150 mg), tramadol (75 mg), acetaminophen (450 mg)
Patient F	Nutritional polyneuropathy	M	43	16.9	4	10	8	100	Pregabalin (600 mg), duloxetine (40 mg), tramadol (150 mg), mirtazapine (15 mg)
Patient G	Lumbar adhesive arachnoiditis	F	74	26.2	2	8	8	15	Pregabalin (75 mg), tramadol (100 mg)
Patient H	Lumbar adhesive arachnoiditis	F	76	26.1	1	5	6	0	Pregabalin (125 mg), NSAIDs (as needed)
Patient I	Chemotherapy-induced peripheral neuropathy	F	81	30.0	10.5	5	5	16	Duloxetine (40 mg)
Patient J	Lumbar adhesive arachnoiditis	F	64	22.9	13	1	1	16	Pregabalin (75 mg)
Patient K	Lumbar radiculopathy	F	64	22.0	7	5	4	18	NSAIDs (as needed)
Patient L	Lumbar radiculopathy and cervical myelopathy	F	51	21.1	2.7	5	5	19	Gabapentin (2400 mg), NSAIDs (as needed)
Patient M	Lumbar radiculopathy and cervical myelopathy	M	73	26.6	2.5	7	7	80	Tramadol (150 mg)
Patient N	Lumbar radiculopathy	F	53	26.4	0.1	10	3	48	Pregabalin (150 mg)
Patient O	Lumbar adhesive arachnoiditis	M	84	21.9	8	8	6	35	Pregabalin (225 mg), tramadol (225 mg), acetaminophen (1350 mg)
Patient P	Thoracic myelitis	M	48	32.5	2.7	10	8	80	Pregabalin (600 mg), amitriptyline (50 mg), NSAIDs (as needed)
Patient Q	Lumbar adhesive arachnoiditis	F	80	21.7	3.5	9	7	21	Pregabalin (75 mg), duloxetine (60 mg)
Patient R	Post-herpetic neuralgia	F	68	22.4	0.1	4	2	26	Pregabalin (100 mg), NSAIDs (as needed)
	Mean (SD)		67.2 (11.7)	23.9 (3.8)	4.7 (3.8)	6.8 (2.6)	5.4 (2.1)	39.8 (29.3)	

Data are expressed as mean (standard deviation [SD]).

NRS, numeric rating scale; NPSI, Neuropathic Pain Symptom Inventory; NSAIDs, non-steroidal anti- inflammatory drugs.

**Table 2:** Relationships of pain scores and laboratory measurements between female and male.

	Female (n = 11)	Male (n = 7)	p value
Age (years)	68.0 (11.0)	70.0 (16.0)	0.856
NRS max	5.0 (3.5)	8.0 (2.0)	0.181
NRS ave	5.0 (3.0)	7.0 (1.5)	0.082
NPSI scores			
Burning	3.0 (5.0)	8.0 (4.5)	0.059
Pressing	4.5 (3.3)	8.0 (7.0)	0.317
Paroxysmal	0.5 (1.8)	5.5 (4.3)	0.011
Evoked	1.0 (2.0)	7.7 (6.7)	0.072
Paresthesia/dysesthesia	4.0 (4.3)	8.0 (2.3)	0.006
Total NPSI	19.0 (17.5)	80.0 (44.5)	0.011
HADS ansiety	9.0 (5.0)	10.0 (4.5)	0.746
HADS depression	8.0 (2.0)	8.0 (3.0)	0.679
16:0 LPA (nM)	12.7 (4.0)	15.0 (4.6)	0.021
16:1 LPA (nM)	0.0 (0.3)	0.8 (0.4)	0.025
18:0 LPA (nM)	6.9 (3.0)	9.3 (6.1)	0.246
18:1 LPA (nM)	7.5 (2.1)	9.4 (2.1)	0.011
18:2 LPA (nM)	1.9 (0.7)	2.0 (3.8)	0.076
20:4 LPA (nM)	2.0 (0.3)	3.3 (2.5)	0.007
16:0 LPC (nM)	2.5 (1.2)	2.8 (1.3)	0.188
16:1 LPC (nM)	0.1 (0.0)	0.1 (0.1)	0.252
18:0 LPC (nM)	1.3 (0.4)	1.6 (0.7)	0.121
18:1 LPC (nM)	1.5 (1.0)	2.0 (0.7)	0.204
18:2 LPC (nM)	0.4 (0.3)	0.5 (0.2)	0.747
20:4 LPC (nM)	0.3 (0.1)	0.4 (0.2)	0.094
Total LPA (nM)	31.3 (8.1)	43.2 (9.6)	0.010
Total LPC (nM)	6.5 (2.7)	7.9 (2.7)	0.205
Autotaxin (mg/l)	1.2 (0.1)	1.1 (0.2)	0.425
pNF-H (pg/ml)	190.0 (299.0)	199.0 (811.5)	0.526

Data are expressed as median (interquartile range [IQR]).

**Fig 1:**
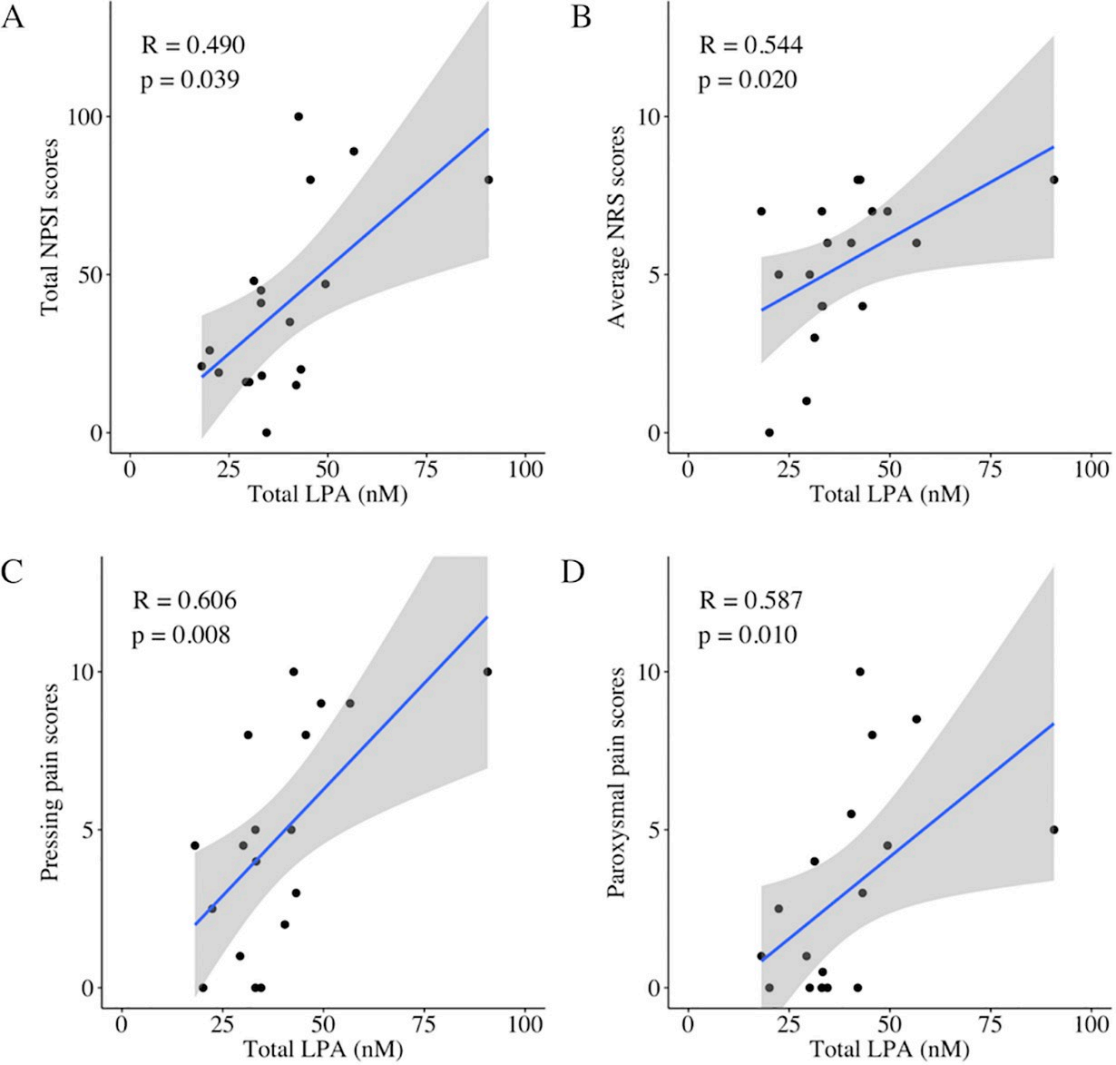
Significant correlations of the total LPA concentrations and pain scores. Total LPA in the cerebrospinal fluid was significantly correlated with total NPSI scores, average NRS scores and two dimensions of the NPSI (pressing and paroxysmal pain). R = correlation coefficient; the grey areas indicate 95% confidence intervals for the regression line.

**Fig 2:**
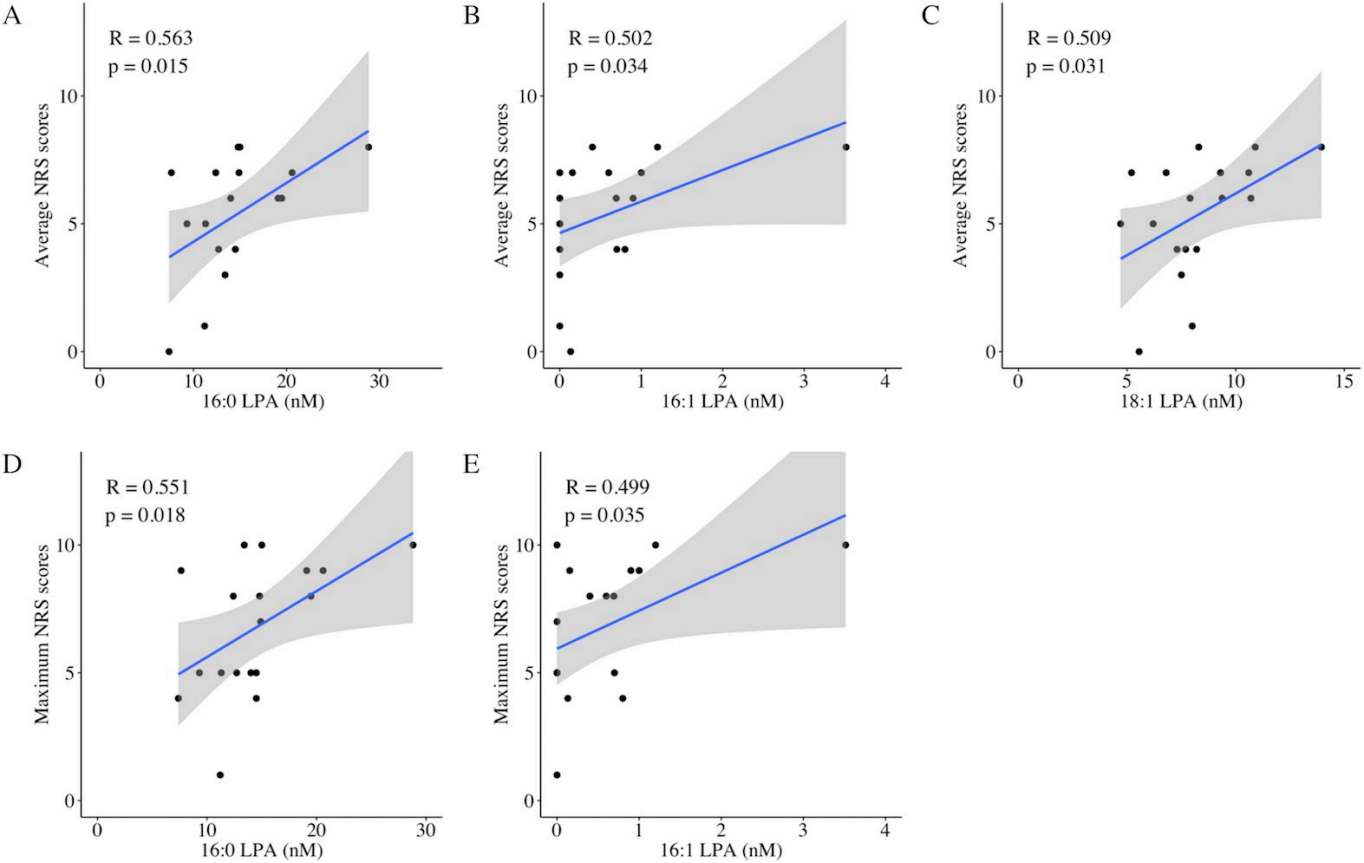
Significant correlations of LPA species (16:0, 16:1 and 18:1 LPA) and NRS scores. The average NRS scores were associated with 16:0, 16:1 and 18:1 LPA (A, B, C), and the maximum NRS scores were associated with and 16:0 and 16:1 LPA (D, E).

**Fig 3:**
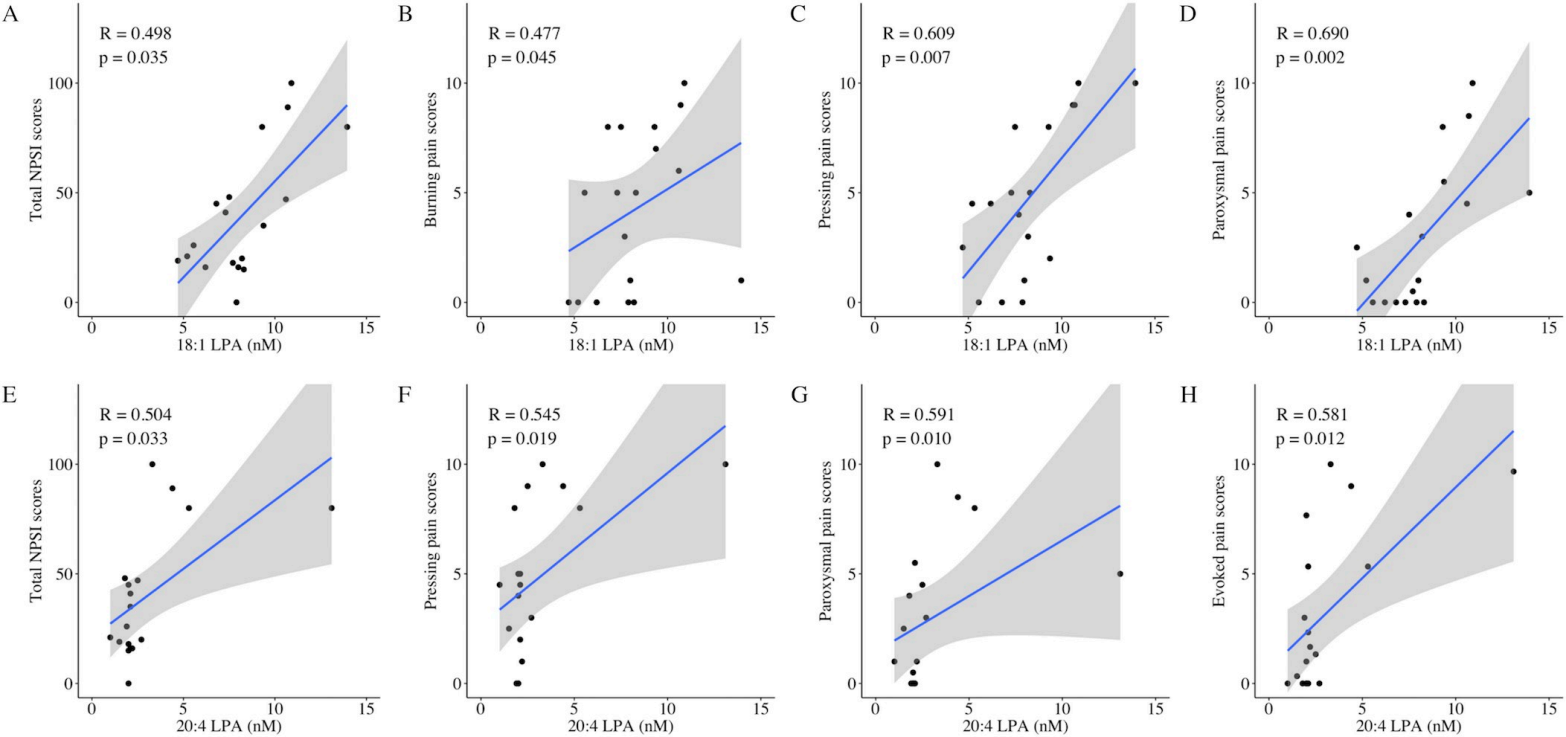
Significant correlations of LPA species (18:1 LPA and 20:4 LPA) and NPSI scores. 18:1 LPA (A, B, C, D) and 20:4 LPA (E, F, G, H) demonstrated the most correlations with 3 dimensions and the total of NPSI scores.

Table 3 shows the results of the Spearman’s correlation analysis investigating the relationship between laboratory measurements (LPA, LPC, autotaxin, and pNF-H) and clinical pain parameters. Regarding molecular species of LPA and LPC, 12 species (14:0, 16:0, 16:1, 18:0, 18:1, 18:2, 18:3, 20:3, 20:4, 20:5, 22:5, and 22:6) were quantified, but only 6 species (16:0, 16:1, 18:0, 18:1, 18:2, and 20:4) were detected in all of the present participants. The total LPA was significantly correlated with total NPSI scores (R = 0.490, p = 0.039), average NRS scores (R = 0.544, p = 0.020), and two dimensions of the NPSI [pressing pain (R = 0.606, p = 0.008); and paroxysmal pain (R = 0.587, p = 0.010)] (Fig 1). In the molecular species of LPA, there were different correlation patterns with pain scores. Four LPA species (16:0, 16:1, 18:1, and 20:4 LPA) were associated with some kinds of pain measurements including the total NPSI. 16:0, 16:1 and 18:1 LPA were associated with the average NRS scores and 16:0 and 16:1 LPA were associated with the maximum NRS scores (Fig 2). Among all the LPA and LPC species, 18:1 and 20:4 LPA demonstrated the most correlations with the NPSI scores [3 dimensions of the NPSI and the total NPSI, respectively] (Fig 3). The other two LPA species (18:0 and 18:2 LPA) were not associated with any pain measurements.

**Table 3:** Correlations between laboratory measurements (LPA, LPC, autotaxin, and pNF-H) and clinical pain parameters.

		pNF-H	Burning	Pressing	Paroxysmal	Evoked	Paresthesia/dysesthesia	Total NPSI	NRS max	NRS ave	HADS anxiety	HADS depression	Age
16:0 LPA	R	0.113	0.451	0.630	0.651	0.210	0.409	0.493	0.551	0.563	0.097	-0.079	-0.150
	p value	0.656	0.060	0.005	0.003	0.403	0.092	0.038	0.018	0.015	0.702	0.755	0.553
16:1 LPA	R	0.320	0.344	0.514	0.425	0.415	0.535	0.584	0.499	0.502	0.165	0.030	-0.192
	p value	0.195	0.162	0.029	0.079	0.087	0.022	0.011	0.035	0.034	0.513	0.905	0.446
18:0 LPA	R	-0.189	0.359	0.305	0.108	0.125	-0.044	0.143	0.124	0.252	0.507	0.010	0.087
	p value	0.453	0.143	0.219	0.669	0.621	0.864	0.573	0.624	0.314	0.032	0.967	0.732
18:1 LPA	R	0.199	0.477	0.609	0.690	0.323	0.450	0.498	0.467	0.509	0.017	-0.084	-0.250
	p value	0.428	0.045	0.007	0.002	0.191	0.061	0.035	0.051	0.031	0.947	0.740	0.317
18:2 LPA	R	0.218	0.217	0.179	0.439	0.258	0.254	0.269	0.052	0.219	-0.163	-0.144	-0.140
	p value	0.385	0.388	0.476	0.068	0.300	0.310	0.281	0.839	0.383	0.519	0.568	0.578
20:4 LPA	R	0.231	0.324	0.545	0.591	0.581	0.417	0.504	0.170	0.332	0.014	-0.013	-0.213
	p value	0.357	0.190	0.019	0.010	0.012	0.085	0.033	0.499	0.178	0.957	0.960	0.396
16:0 LPC	R	0.140	0.152	0.512	0.297	0.447	0.183	0.309	0.096	0.331	-0.095	-0.092	-0.271
	p value	0.580	0.546	0.030	0.232	0.063	0.467	0.213	0.705	0.180	0.708	0.716	0.277
16:1 LPC	R	0.001	0.166	0.547	0.273	0.543	0.247	0.315	0.290	0.349	-0.177	-0.183	-0.634
	p value	0.996	0.511	0.019	0.273	0.020	0.323	0.203	0.244	0.156	0.482	0.467	0.005
18:0 LPC	R	0.220	0.037	0.339	0.358	0.405	0.185	0.263	-0.085	0.077	-0.038	-0.011	-0.134
	p value	0.380	0.884	0.168	0.145	0.095	0.463	0.292	0.738	0.762	0.880	0.965	0.596
18:1 LPC	R	0.088	0.171	0.548	0.211	0.461	0.170	0.308	0.158	0.394	0.034	-0.108	-0.340
	p value	0.728	0.497	0.019	0.401	0.054	0.501	0.214	0.530	0.106	0.892	0.669	0.167
18:2 LPC	R	0.208	-0.220	0.218	-0.022	0.384	-0.118	-0.042	-0.174	0.144	-0.125	0.095	-0.177
	p value	0.407	0.380	0.385	0.931	0.115	0.642	0.870	0.489	0.570	0.620	0.707	0.482
20:4 LPC	R	0.194	0.183	0.559	0.288	0.561	0.265	0.393	0.175	0.409	-0.057	-0.118	-0.363
	p value	0.441	0.466	0.016	0.246	0.015	0.287	0.107	0.487	0.092	0.821	0.640	0.138
Total LPA	R	0.060	0.398	0.606	0.587	0.313	0.388	0.490	0.424	0.544	0.077	-0.131	-0.172
	p value	0.813	0.102	0.008	0.010	0.206	0.111	0.039	0.079	0.020	0.762	0.604	0.494
Total LPC	R	0.110	0.124	0.525	0.277	0.426	0.160	0.293	0.090	0.346	-0.092	-0.140	-0.295
	p value	0.665	0.625	0.025	0.266	0.078	0.526	0.239	0.721	0.160	0.717	0.579	0.234
Autotaxin	R	-0.218	-0.242	-0.320	-0.408	0.040	-0.351	-0.340	-0.437	-0.386	0.458	0.196	0.105
	p value	0.385	0.333	0.195	0.093	0.875	0.153	0.168	0.070	0.113	0.056	0.435	0.677
pNF-H	R	1.000	0.028	0.089	-0.031	0.343	0.195	0.130	0.144	0.362	-0.104	0.380	0.268
	p value		0.911	0.726	0.904	0.164	0.438	0.607	0.569	0.140	0.680	0.120	0.283
Age	R	0.268	-0.140	-0.390	-0.402	-0.333	-0.354	-0.363	-0.164	0.104	0.413	0.660	1.000
	p value	0.283	0.579	0.109	0.098	0.177	0.150	0.139	0.517	0.683	0.089	0.003	

Gray shadows indicate statistically significant correlations (p < 0.05).

Burning, Pressing, Paroxysmal, Evoked, Paresthesia/Dysesthesia and Total NPSI indicate NPSI dimensions of “burning (superficial) spontaneous pain”, “pressing (deep) spontaneous pain”, “paroxysmal pain”, “evoked pain”, “paresthesia/dysesthesia”, and the total scores of these dimensions, respectively. NRS max and NRS ave indicate the maximum and the average NRS scores. LPC, lysophosphatidylcholine; LPA, lysophosphatidic acids; pNF-H, phosphorylated neurofilament heavy subunit; Hospital Anxiety and Depression Scale, HADS.

**Fig 4:**
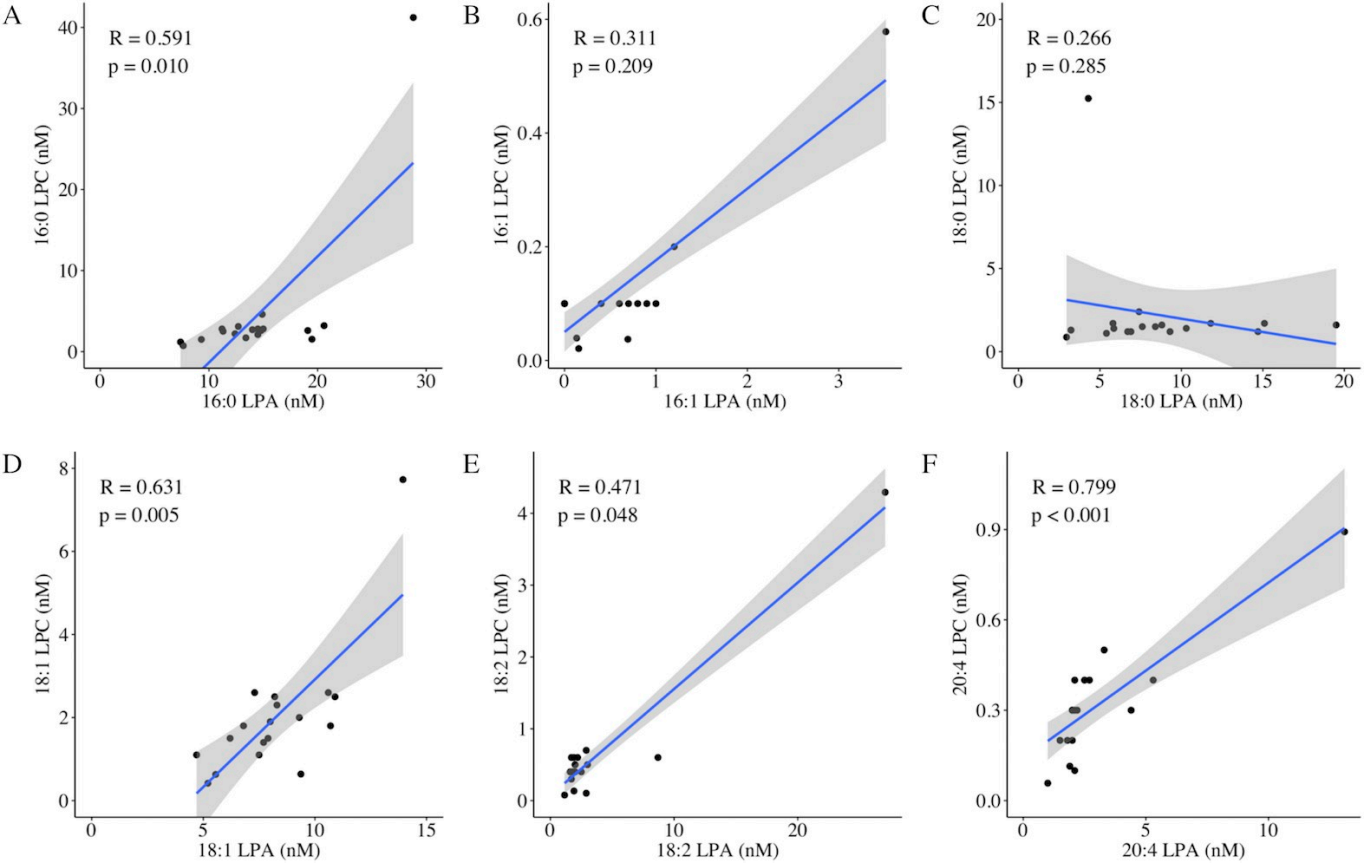
Correlations of CSF concentrations of LPA species with LPC species (16:0, 16:1, 18:0, 18:1, 18:2, 20:4). Four of the six correspondent species of LPA and LPC (16:0, 18:1, 18:2, and 20:4) showed a significant correlation (A, D, E, F). R = correlation coefficient; the grey areas indicate 95% confidence intervals for the regression line.

Four of the six correspondent species of LPA and LPC (16:0, 18:1, 18:2, and 20:4) showed a significant correlation (Fig 4). As for the LPC species, most of them (16:0, 16:1, 18:1, 18:2, and 20:4 LPC) exhibited a relationship with different pain measurements. However, the total LPC was not associated with any pain scores, except for the pressing pain scores. Autotaxin was entirely unrelated to any laboratory measurements and any pain symptoms. Furthermore, pNF-H showed no association with any LPA and LPC measurements or clinical parameters. Mental status (anxiety and depression) showed no correlation with the laboratory measurements except 18:0 LPA.

## Discussion

Average pain intensity and total NPSI scores were linearly correlated with the total amount of LPA. Regarding the molecular species of LPA, 16:0, 16:1 and 18:1 LPA were correlated with average pain intensity, but the other LPA species were not. Different LPA molecular species showed correlations with different dimensions of the NPSI. Most of the correspondent species of LPA and LPC (16:0, 18:1, 18:2, and 20:4) were correlated with one another. Some LPC species showed correlations with NPSI dimensions. Autotaxin and pNF-H showed no correlations with LPA, LPC and clinical measurements.

Our findings indicate that LPA is profoundly associated with neuropathic pain in human subjects, which follows previous findings from animal neuropathic pain models [9]. Focusing on the linear correlations between the CSF LPA and LPC species, LPA concentrations were relatively higher than LPC concentrations (Fig 4). This conflicts with findings that serum LPA concentrations were lower than serum LPC concentrations in patients with acute coronary syndrome [21]. Possibly because LPA is derived from LPC via the enzymatic activity of autotaxin and autotaxin is rich in the CSF [22], LPA might be produced efficiently in the CSF. Despite previous findings that nerve damage can increase LPC [12], the total amount of LPC and autotaxin were not associated with pain intensity in this study. Therefore, LPC and autotaxin by themselves may not have the potential to induce neuropathic pain, but may simply mediate the induction of neuropathic pain through LPA production [10].

We found that four of the measured LPA species, but not 18:0 and 18:2 LPA, were associated with the total NPSI score and the NPSI dimensions. Different LPA species activate LPA receptor subtypes with varied efficacies [11]. Among the LPA receptor subtypes, LPA1 and auxiliary LPA3 are known to play a crucial role in demyelination and neuropathic pain [8]. 18:1 LPA demonstrated the most correlations with neuropathic pain measurements (average pain intensities, the total NPSI score, and 3 out of 5 NPSI dimensions). The 18:1 LPA molecular species has been revealed as key in animal neuropathic pain models, and is the ligand predominantly responsible for LPA1 and LPA3 receptor-mediated amplification of LPA production through microglial activation [13]. Other than 18:1 LPA, 16:0, 16:1 and 20:4 LPA demonstrated correlations with some dimensions of the NPSI. These LPA species have been reported to have a relatively high agonist potency on LPA1 and LPA3 receptors, secondary to 18:1 LPA, in a neuropathic pain animal model [13]. Supporting evidence has been derived from other disease conditions, such as fibrosis and cancer, in which the pathogenicity of the LPA1 receptor signaling is potent in order of 18:1, 20:4, and 16:0 LPA [23]. The agonist potencies of both 18:0 LPA on LPA1 and LPA3 and 18:2 LPA on LPA1 have been reported to be relatively low according to calcium mobilization assay [13], and possibly thereby 18:0 LPA and 18:2 LPA did not show any correlations with neuropathic pain in this study.

The axonal damage biomarker, pNF-H, was not correlated with any LPA or LPC species in the present study. LPA1 receptor activation reportedly mediates the downregulation of myelin proteins, and nerve injury-induced downregulation of myelin proteins leads to the demyelination of the peripheral nerve [24, 25]. In contrast, LPA activation could upregulate axonal growth followed by central sprouting of myelinated fibers in the spinal dorsal horn [26]. Such contradictory effects of LPA on myelin and axons might explain the present pNF-H results.

With respect to the nature of pain (i.e., different dimensions of the NPSI), patients with certain pain syndromes frequently select characteristic words to describe their pain. For example, cancer pain patients consistently characterize their pain as shooting, sharp, dull, and heavy [27], whereas patients with neuropathic pain tend to describe their pain as burning, shooting, tingling, and piercing [28]. We previously demonstrated that one of the two categories of neuropathic pain descriptions are more responsive to mirror visual feedback treatment than the other category [29]. Thus, the nature of pain is considered useful for understanding the underlying pathophysiological mechanisms of pain and forecasting analgesic responses to treatment [30]. In the present study, different LPA and LPC species were associated with different dimensions of the NPSI. Therefore, LPA and maybe LPC species might have respective pathophysiological mechanisms of neuropathic pain. Among the NPSI dimensions, only superficial spontaneous pain (i.e., burning pain) was weakly correlated with 18:1 LPA only. Burning pain is a major characteristic of neuropathic pain, and so future work should explore other LPA and LPC species or other explanatory mechanisms that could be linked to burning pain.

We investigated the influence of other factors such as gender and age on the LPA concentrations and pain symptoms. Table 2 demonstrated that male patients showed higher LPA concentrations than female. Male also had higher pain scores than female, which would affect the high levels of LPA concentrations in male patients. Regarding the factor of age, this study demonstrated age was not associated with either LPA concentrations or pain scores as shown in Table 3. Possible confounding factors other than gender and age (e.g. types and duration of diseases) might underlie the significant relationships of LPA concentrations and pain scores. However, this is an exploratory study and the sample size was relatively small. Therefore, further research is required to provide more detailed analysis and elucidate the exact mechanism of LPA and chronic pain.

In this study, we revealed for the first time that LPA concentrations are significantly correlated with clinical symptoms in patients with neuropathic pain. This supports findings from basic investigations that LPA signaling is the definitive mechanism of neuropathic pain. Autotaxin is reportedly involved in the development of neuropathic pain; however, we found no relationship between autotaxin and neuropathic pain symptoms.

## Supporting information

S1 Appendix The dataset provided in this study. (XLS)
